# Optimizing lens and organ dose evaluation in head CT examinations using monte carlo simulation: influence of gantry tilt and scan range

**DOI:** 10.1007/s12194-025-00980-5

**Published:** 2025-12-15

**Authors:** Yasushi Katsunuma, Kaoru Sato, Takayuki Hasegawa, Yusuke Koba

**Affiliations:** 1 Tokai University Hospital, 143 Shimokasuya, Isehara, Kanagawa 259-1193 Japan; 2 Japan Atomic Energy Agency, 2-4 Shirakata, Tokai-mura, Naka-gun, Ibaraki, 319-1195 Japan; 3 Tokai University Hachioji Hospital, 1838 Ishikawa-machi, Hachioji, Tokyo 192-0032 Japan; 4 National Institutes for Quantum and Radiological Science and Technology, 4-9-1 Anagawa, Inage-ku, Chiba-shi, Chiba, 263-8555 Japan

**Keywords:** Eye lens dose, Head CT, Gantry tilt, Monte Carlo simulation, Effective dose

## Abstract

To minimize radiation exposure to the eye lens during head computed tomography examinations, we performed a high-precision Monte Carlo simulation using the particle and heavy ion transport code system to investigate the effects of gantry tilt angle and scan range on the dose to the lens and other radiosensitive organs. Utilizing a source model with a 2 mm beam width, we visualized the sharp dose peak and the subsequent gradually attenuating tail in the dose distribution to the lens, quantitatively demonstrating that even minor adjustments in the tilt angle or slice position can markedly impact the lens dose. The tilt angle was defined relative to the orbitomeatal line; an upward tilt of +5˚ or more reduced the lens dose by up to 87%. Additionally, a -35˚ downward tilt substantially reduced the lens dose, which is attributed to the presence of high-attenuation tissues in the x-ray path. For organs such as the salivary glands, thyroid, and oral mucosa, scan range adjustments affect the effective dose, with up to a 1.5-fold difference observed depending on whether the eyeballs were included in the scan. These findings underscore the critical importance of a precise protocol design, including the gantry tilt and slice range, in minimizing radiation exposure to multiple organs and provide valuable insights into radiation protection and protocol optimization.

## Introduction

With the advancement of multi-slice computed tomography (CT) technology, rapid and high-resolution image acquisition has become feasible, greatly enhancing the diagnostic accuracy and efficiency in clinical practice. However, the growing frequency of examinations has raised international concerns regarding increased radiation exposure in patients [1]. In particular, the unintended irradiation of nontarget organs, most notably the eye lens, which is directly associated with the risk of radiation-induced cataracts, must be minimized as much as possible.

In 2011, the International Commission on Radiological Protection (ICRP) revised its understanding of radiation-induced cataract genesis and concluded that cataracts can occur at significantly lower doses than previously assumed. Consequently, the threshold dose for deterministic effects on the lens was reduced to 0.5 Gy [2]. These revisions have led to the reclassification of the lens as one of the most radiosensitive organs, and greater attention is now required to minimize lens exposure in diagnostic imaging.

Yeoman et al. reported in an international survey that appropriate gantry tilting can reduce the lens dose by up to 87% but that only 32% of the surveyed facilities routinely implemented eye-sparing techniques, revealing insufficient protective measures in clinical settings [3]. Similarly, Harbron et al. analyzed 668 head CT examinations and found that the eyeballs and lenses were included in the scan range in 88% and 57% of cases, respectively [4], indicating that an awareness of lens dose reduction remains inadequate in actual clinical practice.

They further reported that the lens dose can differ by a factor of up to 15 depending on the scan range. However, this finding is based on evaluations using the National Cancer Institute dosimetry system for CT (NCICT) under vertical beam incidence conditions without accounting for factors such as gantry tilt, which are present in actual clinical imaging. Similarly, other widely used organ dose estimation tools, such as WAZA-ARI and VirtualDose, do not support the specification of gantry tilt angles. As the irradiated volume changes significantly depending on whether the scan range includes the eyes, simple dose comparisons across different settings are unreliable.

Several technical approaches have been proposed to reduce the lens dose, including (1) iterative reconstruction algorithms, (2) bismuth shielding, (3) organ-based tube current modulation, and (4) gantry tilting [5]. Among these, physically tilting the gantry of the CT scanner upward by approximately 10˚–12˚ from the orbitomeatal (OM) line has gained attention as an effective method for avoiding direct irradiation of the eye. Consequently, gantry tilting has been incorporated into the European guidelines on quality criteria for CT [6]. Furthermore, several reports, including Nikupaavo et al. [7], have noted that the guidelines recommend a tilt of approximately 10–12° above the OM line to minimize lens exposure. By optimizing clinical protocols, these technical measures can be applied more effectively, allowing for a further reduction in patient radiation exposure.

Traditionally, lens dose evaluations have relied on point dose measurements using thermoluminescent dosimeters (TLDs) or direct measurements using physical phantoms. However, these methods are inherently limited in their ability to evaluate the spatial dose distribution affected by the gantry tilt and scan range settings. TLDs are confined to local point measurements, and phantom-based experiments are not well-suited for accurately and systematically reproducing or comparing complex conditions involving gantry tilting and scan-range variations. These constraints pose significant challenges to performing comprehensive assessments of dose distributions to the lens.

To facilitate more comprehensive quantitative evaluations of organ doses, several dose assessment tools based on Monte Carlo simulation—such as NCICT [8], VirtualDose [9], and WAZA-ARI version 2 [10]—have been developed, allowing for approximate estimations of absorbed and effective doses.

However, most of these tools employ source models with beam widths of 5–10 mm [11, 12], which are insufficient for accurately estimating the dose to small organs such as the eye lens. While they provide some flexibility in defining the scan range, they generally do not allow for fine adjustments to the acquisition parameters, such as the gantry tilt angle, and their calculations are constrained by fixed irradiation conditions embedded within the software. Notably, the term “beam width (5–10 mm)” in the context of Monte Carlo simulations refers to the z-direction thickness of the virtual source model, and it differs both physically and conceptually from the beam collimation width used in clinical CT (e.g., 40 mm or 160 mm). This parameter was defined to improve the spatial resolution of the dose distribution in the simulation and did not directly correspond to the clinical scan conditions.

In recent years, as international attention has increasingly focused on reducing the lens dose in head CT, the limitations of conventional dose evaluation tools have become more evident. These tools lack the capacity to assess dose distributions with high precision for small, radiosensitive organs and cannot flexibly accommodate changes in imaging parameters. In particular, they are unable to simulate detailed gantry tilt adjustments or capture spatial dose distributions in fine anatomical regions, making them inadequate for evaluating strategies to optimize patient radiation doses.

In this study, we developed a novel x-ray source model for CT imaging that enables the free configuration of arbitrary gantry tilt angles. A high-resolution beam setting with a z-direction width of 2 mm was employed, allowing for a detailed quantitative evaluation of the dose distributions in head CT using the particle and heavy ion transport code system (PHITS) Monte Carlo simulation code. The use of a narrow beam width improved the spatial resolution, enabling the precise characterization of dose variations within individual organs.

This approach made it possible to not only rigorously assess the impact of the gantry tilt and scan range on the lens dose—an analysis that conventional tools are unable to perform—but also visualize the detailed structural features of the dose distribution, such as sharp peaks in localized regions and gradually attenuating tails. These spatial patterns, including their heterogeneity within small anatomical areas, are difficult to capture using conventional measurement methods, such as the TLD method.

The aim of this study was to provide insights that contribute to optimizing the lens dose and rational design of scanning protocols based on such high-precision evaluations.

## Methods

### Monte carlo simulation using PHITS

#### X-ray source model for CT Scanner

An x-ray source model for CT imaging was implemented using the PHITS Monte Carlo code (version 3.24) [13]. The Siemens SOMATOM Definition Flash scanner (120 kV tube voltage), also adopted in the WAZA-ARI system [11, 14–16], was selected as the reference model. The x-ray spectrum and bowtie filter data were defined in the PHITS subroutine “usrsors.” To incorporate gantry tilt variations, the incident beam direction was adjusted using the [transformed] section of PHITS. A beam width of 2 mm was applied to enable high-resolution dose evaluations for small organs such as the eye lens.

#### Phantom model

An adult female voxel phantom (JF-103), developed by the Japan Atomic Energy Agency, was used in this study [17]. This phantom was constructed based on the average height, weight, and organ/tissue masses of adult Japanese females. The voxel size is 0.98 × 0.98 × 1 cm^3^.

It conforms to the ICRP organ classification system and incorporates tissue composition and density information as defined in ICRU reports, making it well-suited for performing an accurate radiation dose assessment.

#### Simulation conditions

**Fig. 1:**
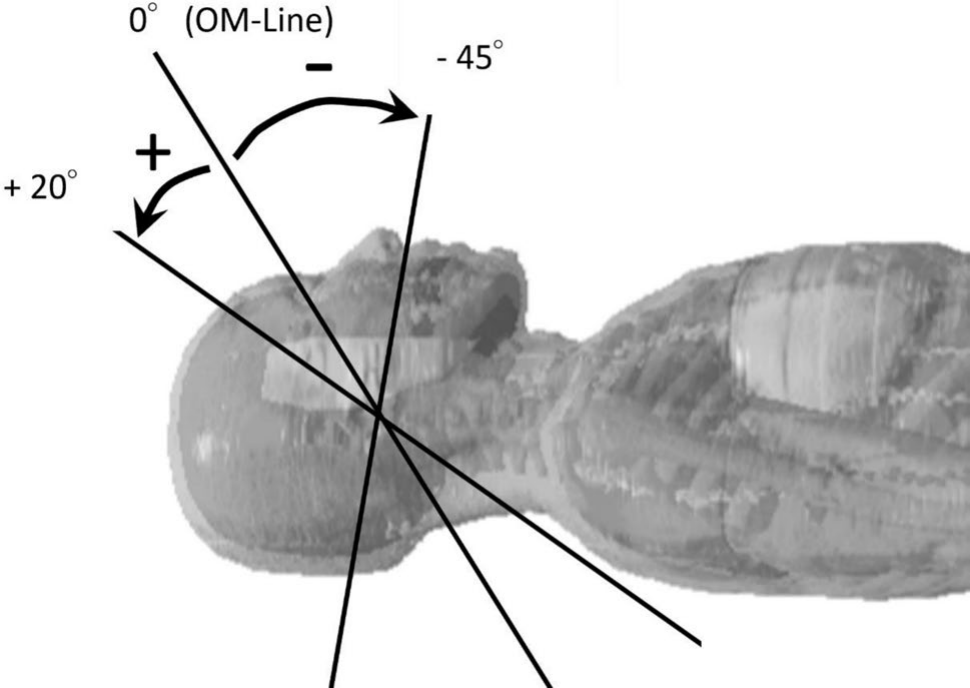
Gantry Tilt Angles Based on the OM Line. The orbitomeatal (OM) line was defined as the reference angle (0˚), with the x-ray beam incidence tilted upward to a maximum angle of +20˚ and downward to a maximum angle of -45˚.

To simulate head CT imaging with varying gantry tilt angles, the OM line was defined as the reference position at 0° (Fig. 1), and the gantry tilt was varied from − 45° to +20° in 5° increments. For each angle, the incident direction of the x-ray beam in the source model was adjusted accordingly.

These tilt angles do not correspond directly to the physical gantry angles of the CT systems; rather, they represent geometrical variations in the beam incidence direction relative to the phantom position, assuming head extension or flexion and with the OM line as the baseline. In clinical settings, most CT scanners are capable of physical gantry tilts of approximately ±30°, and even greater effective tilt angles can be achieved through patient positioning adjustments (e.g., neck flexion or extension). Therefore, the tilt angles from -45° to +20° used in this study were deemed clinically reproducible.

Two scan ranges were defined to evaluate the effects of gantry tilt and scan coverage on the organ dose.

- ・Scan range (i): brain and eyeballs
- ・Scan range (ii): brain only

The cutoff energies for the radiation transport calculations were set to 1 keV for photons and 10 keV for electrons. The number of incident photons was adjusted such that the statistical uncertainty of energy deposition in the major organs remained less than 5%.

#### Dose calculation

The absorbed dose *D(T,k)* [mGy] to organ *T* at slice position *k* was calculated using the following equation:

$${\boldsymbol{D}}\left({\boldsymbol{T}},{\boldsymbol{k}}\right)=\frac{{\boldsymbol{q}}{\boldsymbol{D}}({\boldsymbol{T}},{\boldsymbol{k}})}{{\boldsymbol{q}}{\boldsymbol{K}}\mathbf{a}\mathbf{i}\mathbf{r}}\times \mathbf{n}{\mathbf{C}\mathbf{T}\mathbf{D}\mathbf{I}}_{\mathbf{a}\mathbf{i}\mathbf{r}}\times \frac{{\boldsymbol{A}}{\boldsymbol{t}}}{{\boldsymbol{p}}{\boldsymbol{i}}{\boldsymbol{t}}}$$

where:

*qD(T,k)* [mGy/photon] is the absorbed dose to organ *T* per emitted photon,

*qK*_*air*_ [mGy/photon] is the air kerma per photon at the isocenter,

*nCTDI*_*air*_ is the CTDI _free air_ per mAs (0.192 in this study),

*At[mAs]* is the product of the tube current [mA] and rotation time [s], and

*pit* is the beam pitch (set to 1.0 in this study).

CTDI-related parameters were based on the “Dose and Image Quality Report” provided by Siemens. The values were set as follows: *nCTDI*_*air*_ = 0.192, pitch (*pit*) = 1.0, and rotation time = 1.0 s.

Furthermore, to comply with the diagnostic reference level for head CT examinations recommended by the American College of Radiology (ACR)[18] (i.e., CTDI_vol_ = 75 mGy), the tube current-time product (*At*) was calculated using the WAZA-ARI (version 2) dose evaluation web tool[10]. Based on the scan conditions assumed in this study (e.g., scan range and beam quality), the *At* value required to achieve a CTDI_vol_ of 75 mGy was determined to be 540 mA (tube current: 540 mA, rotation time: 1.0 s).

This setting was not derived from direct measurements but was established through simulation for the purpose of aligning with the ACR reference level.

Based on the absorbed dose *D(T, k)* [mGy] at each slice position k, dose distribution profiles were constructed for the lens, eyeballs, and brain. As shown in Fig. 3, the total absorbed dose for each organ was calculated by integrating the dose distribution over the scan range defined by lines (i)–(iv), starting from a slice position of 0 mm.

The full width at half maximum (FWHM) was calculated to quantitatively evaluate the width of the dose peak in the lens. The FWHM is defined as the distance between two slice positions where the absorbed dose exceeded half of the maximum value, *D*_*max*_. Specifically, for the dose distribution *D(x)*, the slice positions *x₁* and *x₂* (with *x₁* < *x*_*peak*_ < *x₂*) that satisfy:

$$D\left({x}_{1}\right)=D\left({x}_{2}\right)=\frac{1}{2}{D}_{max}$$

were identified, and the FWHM was computed as:

$$FWHM={x}_{2}-{x}_{1}$$

Based on the dose distribution in the lens shown in Fig. 3, the FWHM was calculated for each gantry tilt angle. For example, at the OM line (0° tilt), the peak value was 18.6 mGy, and the FWHM was defined as the slice range in which the absorbed dose exceeded 9.3 mGy, corresponding to approximately 181–187 mm. This region is considered to represent significant exposure to direct irradiation.

Furthermore, the equivalent doses [mSv] for each organ were obtained by multiplying the absorbed dose [mGy] by a radiation weighting factor of 1.0. The effective dose (*E*_*eff*_) was calculated by applying the tissue weighting factors defined in ICRP Publication 103[19].

### Comparison with the WAZA-ARI organ dose calculation tool

#### Objective of the comparison

In this study, we simulated scan conditions with controlled gantry tilt angles, with and without the inclusion of the eyes, and compared the lens doses to provide a more clinically realistic evaluation.

This comparison was designed to clarify how protocol variations directly influence lens exposure, since even minor differences in scan range or tilt angle can result in large dose discrepancies for small radiosensitive organs such as the lens.

Furthermore, previous dosimetry tools such as NCICT and WAZA-ARI have not incorporated gantry tilt into their evaluations, limiting their ability to address these clinically relevant scenarios.

By explicitly comparing these conditions, this section establishes the rationale for the subsequent dose analyses and discussion on optimizing clinical CT protocols.

#### Configuration and conditions for the comparison

For the comparison, the following conditions were aligned between the two dose calculation methods.

- Gantry tilt angle: –35˚, defined relative to the OM line in this study. For the JF-103 phantom, this corresponds to the perpendicular beam incidence. Because the WAZA-ARI also assumes a perpendicular incidence to the phantom, this condition ensured consistency.
- In this study, the beam widths of the x-ray source model were 5 mm and 2 mm.
- Scan range:
  o In the WAZA-ARI, the “lower orbital margin” (scan range iii) and “upper orbital margin” (scan range iv) were used.
  o In this study, the scan ranges were defined with the gantry tilt applied: “including the eyeballs” (scan range i) and “excluding the eyeballs” (scan range ii).

This approach enabled a direct comparison between conventional dose evaluation methods and the high-precision gantry tilt-controlled simulation conditions employed in this study. In addition to assessing the influence of the gantry tilt angle and scan range on the lens dose, we evaluated the doses to other organs required for an effective dose estimation.

To further clarify how changes in the dose distribution affect the surrounding tissues, organ-specific absorbed doses were calculated for several key organs located in the neck and facial regions, specifically the thyroid gland, salivary glands, and oral mucosa.

## Results

### Dose distributions in the lens, eyeball, and brain (Figs. 2 and 3)

**Fig. 2:**
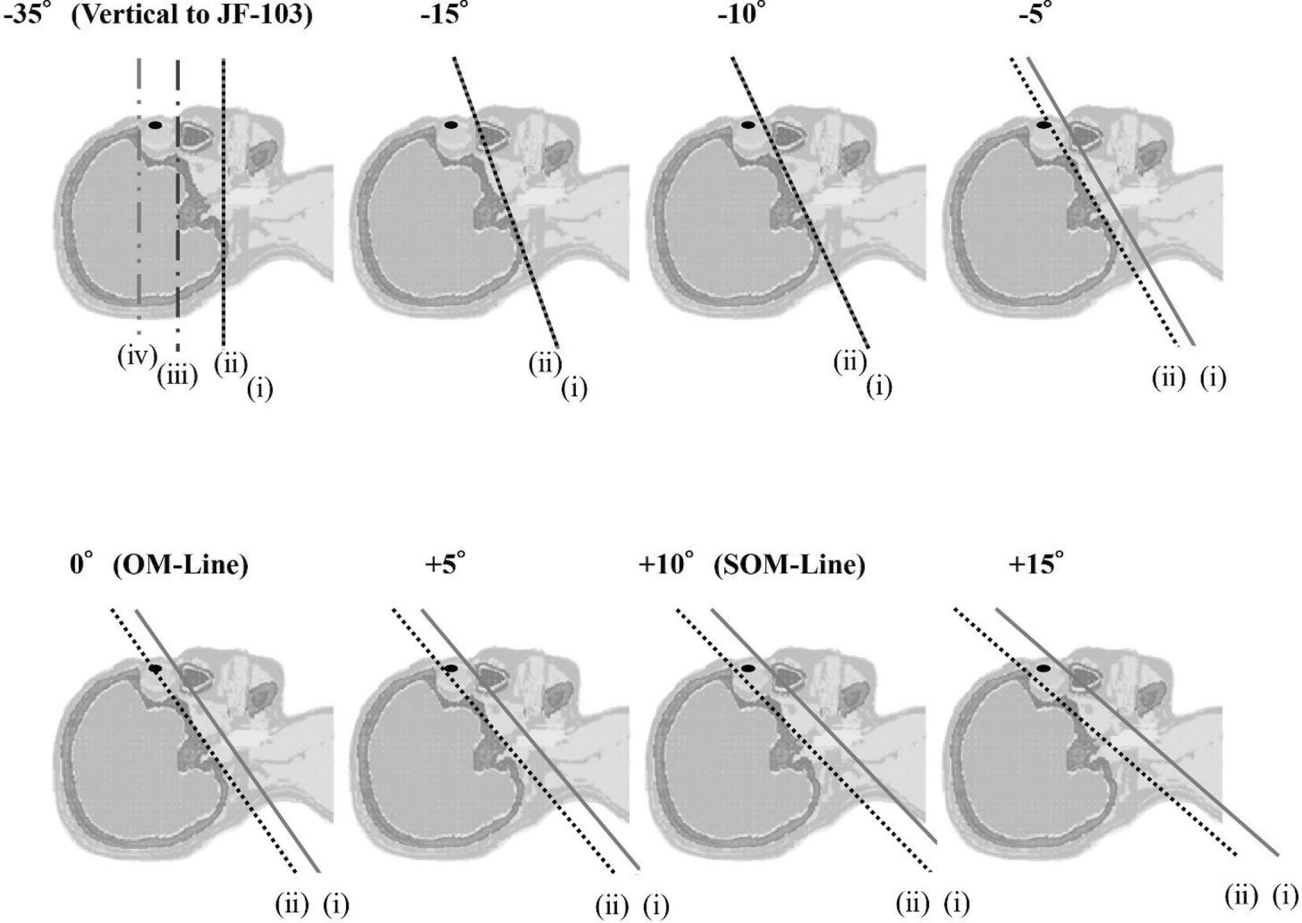
Representative X-ray Beam Incidence Angles and Definitions of the Scan Range. Line (i) indicates the caudal end of the scan range, including both the brain and the eyes, while line (ii) represents the caudal end of the scan range, including the brain only. In contrast, existing organ dose calculation tools (e.g., National Cancer Institute dosimetry system for CT, WAZA-ARI, VirtualDose) typically assume vertical x-ray incidence to the phantom, under which the caudal scan limits are defined as line (iii) (infraorbital margin) and line (iv) (supraorbital margin), respectively.

**Fig. 3:**
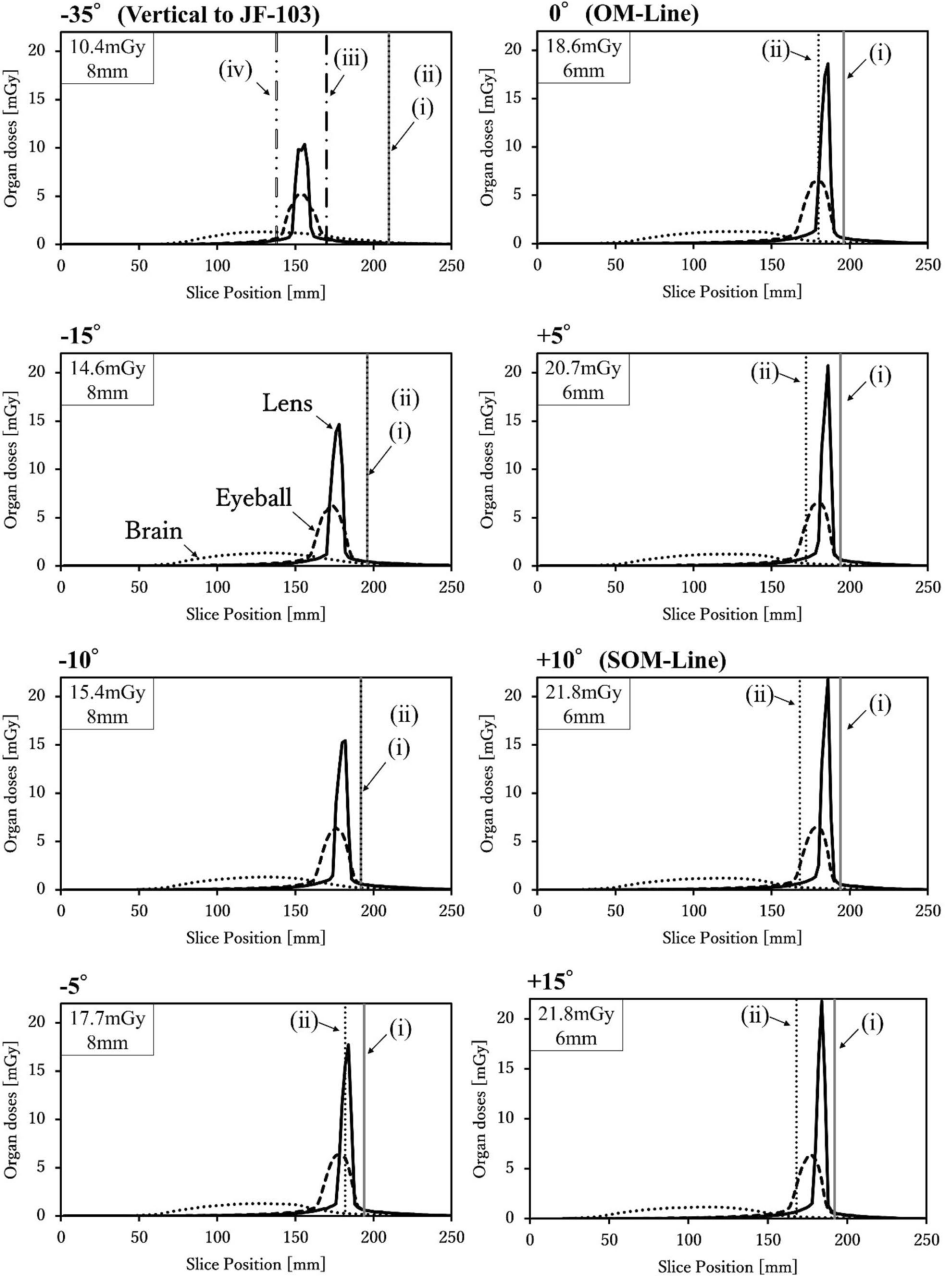
Dose Distributions in the Lens, Eyeball, and Brain at Various X-ray Beam Incidence Angles. This figure depicts the absorbed dose distributions in the lens, eyeball, and brain at each slice position, calculated using a 2 mm beam-width source model covering from the cranial top (0 mm) to 260 mm caudally. Line (i) indicates the caudal end of the scan range including both the brain and eyes, and line (ii) represents that including the brain only. Line (iii) and line (iv) correspond to the infraorbital margin and supraorbital margin, respectively. The maximum absorbed dose in the lens and its full width at half maximum are annotated in the upper left corner of each graph.

Figure 3 depicts the slice-by-slice absorbed dose distributions of the lens, eyeballs, and brain at each gantry tilt angle. The tilt angles shown in Fig. 3 correspond to the representative x-ray beam incidence directions and scan range definitions illustrated in Fig. 2.

In the dose distribution for the lens, a characteristic pattern was observed between the slice positions of 150 mm and 200 mm: a sharp peak surrounded by gradually tapering shoulders. This peak became particularly prominent at gantry tilt angles of +5° and greater, with a peak value of 20.7 mGy at +5°, and a consistently high value of 21.8 mGy at both +10° and +15°. These peaks indicate a localized concentration of the radiation dose when the lens is positioned near the center of the x-ray beam.

In addition, the FWHM was used as an index to assess the spatial extent of the high-dose region (dose concentration) around the lens peak. The FWHM values for each gantry tilt angle are annotated within the dose distribution graphs in the upper-left panels of Fig. 3.

The FWHM was approximately 8 mm at tilt angles from − 35° to − 5° and approximately 6 mm at 0° and greater. This indicates that at 0° (OM line) or greater, the width of the high-dose region becomes narrower, increasing the risk that even slight shifts in the scan position could inadvertently include this peak region within the scanned volume.

Furthermore, in scan range (ii), the peak portion of the lens dose distribution fell outside the scan field at tilt angles of -5° and greater. However, the shoulder region of distribution (dose tail) remained within the scan range. As the gantry tilt angle increased, the absorbed dose in the tail region gradually decreased.

The dose distribution in the eyeball demonstrated less variation across the gantry tilt angles than that in the lens. However, in scan range (ii), once the gantry tilt angle exceeded -5°, the eyeballs shifted outside the scan field, resulting in a decrease in the dose.

The dose distribution in the brain remained stable regardless of the gantry tilt angle, and no significant variation was observed across different angles.

### Comparison of lens doses by the gantry tilt angle and scan range (Fig. 4, Table 1)

**Fig. 4:**
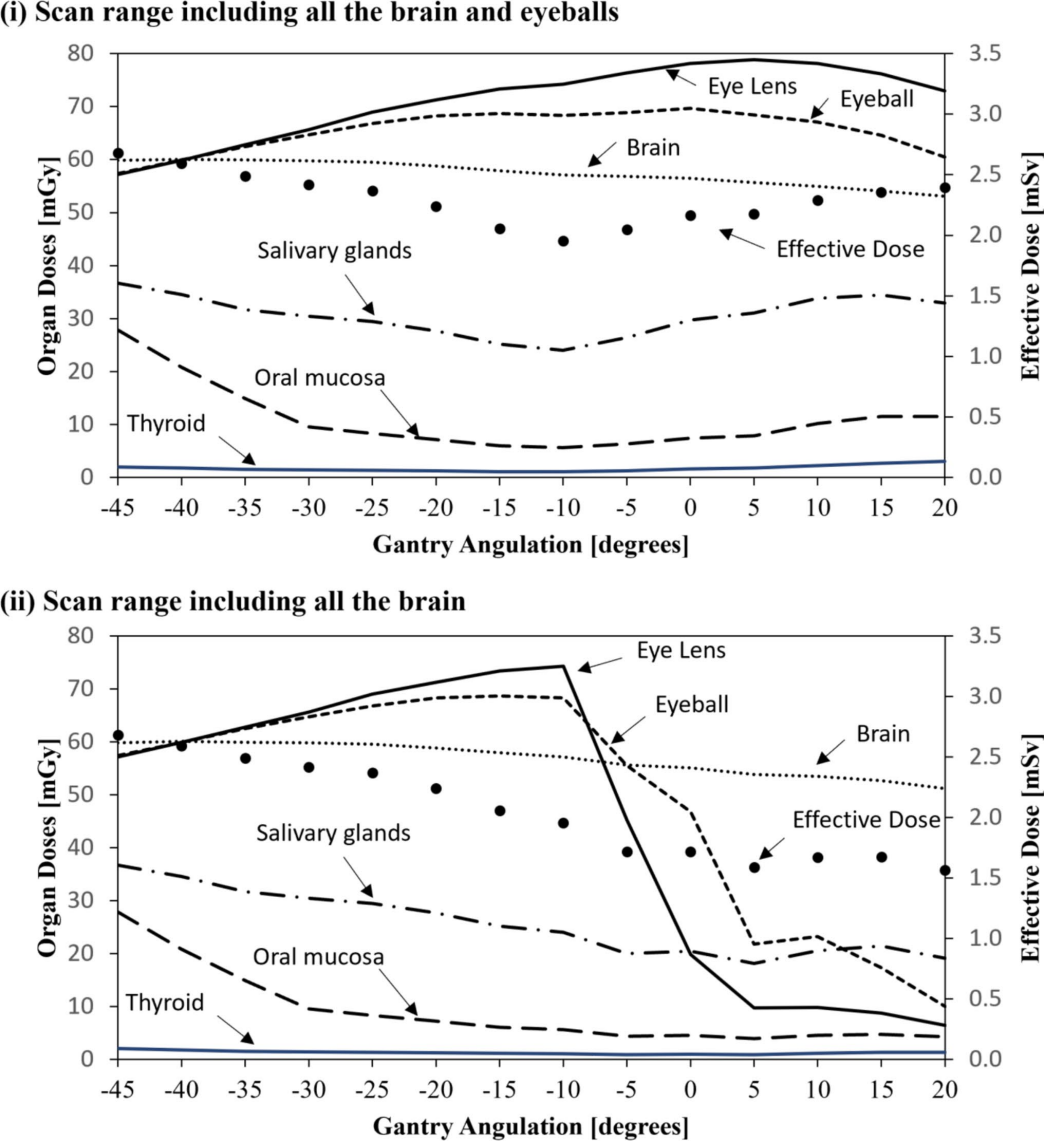
Variations in Organ-Specific Absorbed Doses and Effective Dose According to the Gantry Tilt Angle and Scan Range. This figure illustrates the changes in the absorbed dose (primary axis, mGy) for each organ (lens, eyeball, brain, salivary glands, oral mucosa, and thyroid) and effective dose (secondary axis, mSv) in response to variations in the gantry tilt angle (-45˚ to +20˚). Two scan range conditions are compared: (i) the brain and eyes, and (ii) the brain only. This figure visually summarizes the key findings of this study.

**Table 1:** Organ-Specific Absorbed Doses and Effective Dose According to the Gantry Tilt Angle and Scan Range

	**Lens [mGy]**		**Eyeball [mGy]**		**Brain [mGy]**		**Salivary glands [mGy]**		**Oral Mucosa [mGy]**		**Thyroid [mGy]**		**Effective dose [mSv]**	
**Tilt Angle**	(i)	**(ii)**	**(i)**	**(ii)**	**(i)**	**(ii)**	**(i)**	**(ii)**	**(i)**	**(ii)**	**(i)**	**(ii)**	**(i)**	**(ii)**
**−35˚**	62.8	62.8	62.5	62.5	59.9	59.9	31.7	31.7	14.9	14.9	1.5	1.5	2.49	2.49
**−15˚**	73.3	73.3	68.7	68.7	57.9	57.9	25.2	25.2	6.0	6.0	1.1	1.1	2.06	2.06
**−10˚**	74.3	74.3	68.3	68.3	57.1	57.1	24.0	24.0	5.6	5.6	1.1	1.1	1.95	1.95
**−5˚**	76.4	45.3	68.9	55.5	56.9	55.6	26.4	20.0	6.4	4.4	1.3	0.9	2.05	1.72
**0˚**	78.1	19.8	69.7	46.7	56.4	55.1	29.8	20.5	7.4	4.5	1.6	1.0	2.16	1.72
**+5˚**	78.8	9.7	68.4	21.7	55.7	53.8	31.0	18.1	7.8	3.9	1.8	0.9	2.18	1.59
**+10˚**	78.1	9.8	67.1	23.2	55.0	53.4	33.8	20.5	10.2	4.6	2.3	1.2	2.29	1.67
**+15˚**	76.2	8.8	64.6	17.3	54.1	52.6	34.4	21.4	11.5	4.8	2.7	1.3	2.35	1.67

This table presents the numerical impact of changes in the gantry tilt angle (-35˚ to +15˚) and scan range settings during head CT examinations on the absorbed doses (mGy) for individual organs (lens, eyeball, brain, salivary glands, oral mucosa, and thyroid) and the effective dose (mSv). Two scan range conditions were evaluated: (i) the lens and eyeball, and (ii) the brain only.

The tilt angles of −35°, 0°, and +10° correspond to vertical, OM-line, and SOM-line incidence, respectively.

Figure 4 provides a visual representation of the variation in the lens dose as a function of the gantry tilt angle, whereas Table 1 summarizes the representative absorbed dose values for selected tilt angles (-35° to +15°) at which dose changes were particularly pronounced and clinically relevant, as illustrated in Fig. 4. Together, these allow for both the visual evaluation and quantitative evaluation of variations in the lens dose.

In scan range (i) (including both the brain and eyeballs), the lens dose increased as the gantry tilt increased, increasing from − 45° to +5°, it and reached a maximum value of 78.8 mGy at +5° (Fig. 4). As shown in Table 1, the dose at − 35° (perpendicular beam incidence) was 62.8 mGy, indicating an increase of approximately 26% compared with this reference. At -45°, the dose was 57.2 mGy, representing a 26.9% reduction relative to the dose at 0° (OM line, 78.1 mGy).

In contrast, in scan range (ii) (including the brain only), the lens dose decreased sharply as the gantry tilt angle increased. As described in section 3.1, this was because the peak of the lens dose distribution shifted outside of the scan field at tilt angles of -5° and greater.

At +5°, the lens dose decreased to 9.7 mGy, and at +15°, it decreased further, to 8.8 mGy, corresponding to an 85%–86% reduction compared with − 35° (perpendicular beam incidence). At − 5° (45.3 mGy) and 0° (19.8 mGy), although the peak was excluded from the scan range, the shoulder region of the dose distribution remained partially within the field, resulting in residual dose levels.

### Dose variation in the thyroid, salivary glands, and oral mucosa (Fig. 4, Table 1)

The absorbed doses in the thyroid gland, salivary glands, and oral mucosa varied depending on the gantry tilt angle and scan range. Between − 45˚ and − 10˚, there was little difference between scan ranges (i) and (ii); however, from -5˚ and greater, clear dose discrepancies were observed between the two ranges.

- Thyroid: In scan range (i), the dose increased from 1.3 mGy to a maximum of 3.0 mGy as the tilt angle increased from -5˚ to +20˚. In scan range (ii), the dose ranged from 0.9 mGy to 1.4 mGy.
- Salivary Glands: In scan range (i), the dose increased from 26.4 mGy to 34.4 mGy between -5˚ and +15˚. In scan range (ii), the dose slightly decreased from 20.0 mGy to 19.1 mGy.
- Oral Mucosa: In scan range (i), the dose rose from 6.4 mGy to 11.6 mGy between -5˚ and +15˚, whereas in scan range (ii), it slightly decreased from 4.4 mGy to 4.2 mGy.

### Comparison with conventional dose calculation tools (Figs. 2 and 3, Table 1)

Under the evaluation conditions described by Harbron et al. [4], namely, a comparison between scans including the eyeballs without gantry tilt and those excluding the eyeballs without gantry tilt, the WAZA-ARI tool estimated lens doses of 57.8 mGy for the infraorbital margin (scan range (iii)) and 4.6 mGy for the supraorbital margin (scan range (iv)), demonstrating a difference of approximately 13-fold.

In contrast, when similar scan ranges were reproduced in this study with the gantry tilt applied, the doses at the OM line (0˚) were 78.1 mGy and 19.8 mGy (a four-fold difference), and the doses at the supraorbitomeatal (SOM) line (+10˚) were 78.1 mGy and 9.8 mGy (an eight-fold difference).

Furthermore, for the condition including the eyeballs, the lens dose in this study (78.1 mGy; scan range (i)) was about 35% greater than that estimated by WAZA-ARI (57.8 mGy; scan range (iii)).

For the condition excluding the eyeballs, WAZA-ARI estimated a lens dose of 4.6 mGy (vertical, scan range (iv)), whereas the present study yielded 19.8 mGy at the OM line and 9.8 mGy at the SOM line (both for scan range (ii)).

### Variation in the effective dose (Table 1, Fig. 4)

The effective dose varied depending on the gantry tilt angle and scan range. Between -45˚ and -10˚, there was no significant difference between scan ranges (i) and (ii), and the effective dose ranged from 2.68 mSv to 1.95 mSv.

However, from -5˚ and greater, a clear difference emerged between the two scan ranges. In scan range (i) (including the eyeballs), the effective dose increased at tilt angles of 0˚ and greater, increasing from 2.16 mSv to a maximum of 2.39 mSv. In contrast, in scan range (ii) (excluding the eyeballs), the effective dose decreased under the same conditions, from 1.72 mSv to 1.56 mSv.

## Discussion

### Detailed analysis of the lens dose and protocol optimization strategies

In this study, a high-resolution x-ray source model with a 2 mm beam width was employed to perform a detailed Monte Carlo analysis of the absorbed dose distributions along the longitudinal (slice) direction during CT scanning. This approach enabled clear visualization of steep lens dose peaks and trailing regions (tails) that could not be captured by conventional point measurements or source models with beam widths of 5–10 mm. The results quantitatively demonstrated that even slight adjustments in the slice positioning or gantry tilt angle can have a substantial impact on the lens dose in clinical CT practice.

For example, when the gantry was tilted upward from the OM line (0°) to +5° and +10°, the lens dose increased to 20.7 mGy and 21.8 mGy, respectively. This demonstrates that even small angular changes can produce steep dose gradients (Figure 2, Figure 3, and Table 2). This phenomenon is attributed to the anatomical positioning of the lens near the edge of the imaging field, where slight variations in the beam incidence or slice location can determine whether the lens is directly exposed to primary x-rays, significantly affecting the absorbed dose.

The sharp peaks observed in the lens dose distribution were presumed to result from direct exposure to primary radiation, whereas the trailing regions (dose tails) were mainly influenced by scattered radiation. Therefore, simply excluding the eyeballs from the scan range is insufficient for fully suppressing lens exposure. Precise control of the gantry tilt and scan coverage, including careful selection of slice positioning, is essential for effective dose management.

Notably, even slight changes in the start position or end position of the scan range can cause the dose peak or its trailing region to fall within the scanned volume, potentially resulting in unintended lens exposure. For example, at the OM line (0°), or with a +5° upward tilt, although the peak may be excluded from the scan range, the tail region can remain within the field, leading to an accumulated dose of approximately 10 mGy (Figure 3). This phenomenon, in which residual exposure persists even when the dose peak is excluded, is often overlooked by conventional simplified dose estimation methods and represents an important new consideration in protocol design.

Furthermore, in helical scanning, even when the lens is positioned outside the intended scan range, a risk of unintended exposure due to over-ranging remains, which may result in irradiation of the tail region or spike-like peaks in the dose distribution. Conventional (axial) scanning can be an effective strategy for reducing such unexpected exposure risks. Modern CT systems are equipped with active or dynamic collimators, which are considered effective in reducing the over-ranging exposure along the z-axis. However, several studies report that complete shielding is not achieved, and a small amount of irradiation beyond the scan range may persist [20–22]. Therefore, in the design of scan protocols, it is essential not to rely solely on automated collimator control but rather to incorporate spatial dose distribution considerations to ensure optimal radiation protection.

In addition to the lens, a reduction in the absorbed dose was observed in other organs as the gantry tilt angle increased. For example, when the gantry was tilted from the OM line (0°) to +10°, the thyroid dose decreased from 2.7 mGy to 1.5 mGy (a reduction of approximately 44%), the salivary gland dose decreased from 34.4 mGy to 21.4 mGy (a reduction of approximately 38%), and the oral mucosa dose decreased from 14.9 mGy to 8.8 mGy (a reduction of approximately 41%) (Table 1). These findings demonstrate that fine-tuning of the x-ray beam incidence angle is effective in not only reducing the lens dose but also lowering the exposure to multiple radiosensitive organs, thereby supporting the broader utility of gantry angulation beyond lens protection.

For the lens, a gantry tilt angle of +5° or greater above the OM line, combined with the exclusion of the eyes from the scan range, resulted in a maximum dose reduction of 87%. This finding is consistent with European guidelines, which recommend a tilt of 10°–12° above the OM line for routine head CT examinations [23]. This also aligns with previous reports demonstrating a dose reduction of approximately 75% at tilt angles around 15° [3, 24], thereby supporting the validity and clinical relevance of the present simulation framework.

Although previous studies have reported the effectiveness of gantry tilt and its impact on the lens dose, their evaluations remain limited. For instance, although Harbron et al. [4] and Yeoman et al. [3] presented lens doses corresponding to various tilt angles, they did not provide slice-by-slice dose profiles or three-dimensional visualizations of the dose distribution. Additionally, studies using radiochromic film have demonstrated dose variations depending on the phantom structure and beam position; however, these investigations relied heavily on point-based measurements, making them insufficient for fully achieving spatial dose heterogeneity [25].

In contrast, the Monte Carlo simulation approach employed in this study effectively overcomes the limitations of conventional measurement-based methods. It enables a highly accurate dose evaluation at arbitrary slice positions and beam angles, making it an extremely useful tool for achieving clinically realistic dose optimization in the future.

Furthermore, in the clinical setting, upward tilting of the gantry is often impractical. In this study, under scan conditions that included the eyes, the lens dose increased progressively as the tilt angle shifted from -35° to +5°, with a 26% increase observed from +5° to − 35° (Figure 4). This increase is likely due to the exclusion of attenuating tissues, such as the skin, skull, eyeballs, and brain parenchyma, from the x-ray path and shortening of the attenuation path length, resulting in reduced attenuation of the x-rays. In other words, downward tilt angles (− 5° to − 35°) increased the amount of overlying attenuating tissues along the beam path, thereby enhancing beam attenuation and functioning as an effective dose-reduction strategy.

This strategy, which leverages anatomical structures for radiation protection, represents a novel approach based on a deliberate beam path design, in contrast to the conventional avoidance-based concept of simply excluding radiosensitive tissues from the scan range. This also suggests its potential applicability in clinical cases in which upward gantry tilting is not feasible.

Harbron et al. [4] reported that, without considering gantry tilt, the lens doses were 47 mGy and 3.1 mGy, an approximately 15-fold difference, for scans that included the eyes and those that excluded the eyes, respectively. In the present study, when reproducing similar scan conditions using the WAZA-ARI system, the corresponding lens doses were 57.8 mGy and 4.6 mGy (approximately a 13-fold difference), largely replicating the quantitative trend reported by Harbron et al. This supports the validity of the irradiation model used in this study.

In contrast, under the tilt-controlled conditions of this study, the lens doses were 78.1 mGy and 19.8 mGy at the OM line (0°) and 78.1 mGy and 9.8 mGy at the SOM line (+10°), corresponding to approximately four-fold and eight-fold differences, respectively. These results demonstrate that the difference in the lens dose due to the scan range varies substantially depending on whether a gantry tilt is applied.

These findings suggest that conventional dose assessments that do not account for gantry tilt may overestimate the dose differences between scan ranges, highlighting the importance of dose evaluation methods capable of realistically reproducing clinical conditions.

Furthermore, this study revealed that the lens dose can vary significantly depending on the presence or absence of gantry tilt, even when the scan ranges are identical. Under eye-including conditions, the lens dose in the WAZA-ARI model with vertical beam incidence was 57.8 mGy, whereas it increased to 78.1 mGy (approximately 35% higher) under the tilted conditions of this study (OM and SOM lines). Under eye-excluding conditions, the WAZA-ARI model yielded a dose of 4.6 mGy, whereas the corresponding values in this study were 19.8 mGy (OM line) and 9.8 mGy (SOM line), indicating a substantial increase.

This indicates that beam angulation alters the irradiation pattern around the lens, enabling an assessment of dose heterogeneity that cannot be captured under conventional vertical incidence conditions.

Therefore, even when the scan ranges are identical, the presence or absence of gantry angulation is a critical factor in organ dose assessment. To achieve more realistic and accurate radiation dose evaluations, it is essential to appropriately incorporate angulation conditions.

We also examined the variations in the effective dose and its contributing factors. For example, at a gantry tilt angle of -35° (vertical incidence), radiosensitive organs, such as the salivary glands and oral mucosa, were included within the scan range, resulting in a relatively high effective dose of 2.49 mSv. In contrast, upward angulation gradually excludes these organs from the scan range, reducing the effective dose to approximately 1.95 mSv at − 10°. However, for gantry tilt angles of -5° or greater, the definition of the scan range had a more pronounced impact on the effective dose. In particular, under scan range (i), which included the eyes, organs such as the salivary glands, oral mucosa, and thyroid gland were again included regardless of the angulation, resulting in an increased effective dose of 2.05–2.35 mSv. Conversely, in scan range (ii), which excluded the eyes, these organs were not included, and the effective dose was further reduced to 1.59–1.67 mSv (Fig. 4 and Table 1).

Thus, the effective dose is strongly influenced by not only the gantry tilt angle but also the organs included within the scan range. Therefore, optimizing the combination of gantry angulation and scan coverage is crucial for reducing overall radiation exposure in patients.

Compared with previous studies, the present study enabled a more detailed and comprehensive evaluation of organ doses across different gantry tilt angles, thereby providing valuable insights for revising clinical imaging protocols. Notably, a clear demonstration of how the combination of gantry angulation and the scan range affects not only the eye and lens doses but also those of the thyroid gland, salivary glands, and oral mucosa represents a unique and practical contribution not observed in prior research.

Furthermore, this study quantitatively evaluated the dose reduction effect achieved by excluding specific organs from the scan range in terms of the effective dose, thereby reinforcing the effectiveness of gantry angulation from the perspective of whole-body exposure. These findings underscore the necessity and significance of optimizing imaging protocols to not only reduce radiation to specific organs but also achieve a balanced reduction in doses to other organs and the overall effective dose.

Notably, Yeoman et al. [3] reported an 87% reduction in the lens dose with gantry angulation, whereas no significant change was observed in the extent of artefacts within the posterior fossa. This finding suggests that a substantial dose reduction can be achieved without compromising image quality. Such evidence is crucial for future protocol designs, for which balancing radiation dose reduction and diagnostic performance is of paramount importance.

Furthermore, as noted by Nikupaavo et al. [7] and Tarkiainen et al. [26], practical implementations of the gantry tilt in clinical settings remain limited. This study provides quantitative evidence supporting its effectiveness and offers a scientific basis for promoting its broader clinical adoption. Future directions include the integration of gantry tilt into imaging protocols that balance the field of view and reconstruction accuracy, as well as the personalization of protocols based on patient-specific factors, such as age and body habitus.

Importantly, although the beam width of 2 mm used in this study was a simulation setting to enhance spatial resolution, the observed reduction in lens dose with gantry tilt and the dependence of lens exposure on the inclusion of the eyes in the scan range are universal findings**.** These results remain valid under routine multislice CT conditions with beam widths of 20–40 mm and therefore directly support the clinical practice of excluding the eyes from the scan range to minimize lens exposure.

### Limitations and future perspectives

In this study, the high-precision dose assessment of the lens and other small organs was achieved by employing Monte Carlo simulations combined with a detailed phantom model. However, this study has several limitations.

First, although this study employed a source model from WAZA-ARI, which has been extensively validated through experimental measurements, a direct comparison with conventional measurements using physical phantoms and dosimeters was not performed. Although the Monte Carlo simulation-based dose assessment method is theoretically known for its high reproducibility and accuracy, experimental validation using physical phantoms remains a challenge for verifying its consistency with measured data. Such efforts are expected to further enhance the reliability and validity of simulation results.

In this study, to quantitatively evaluate the impact of the gantry tilt angle on the organ dose, an x-ray source model for CT imaging was constructed using the Monte Carlo simulation code PHITS. Various tilt conditions were simulated by systematically varying the incident beam angle. The tilt angles were defined in 5° increments from − 45° to +20° relative to the OM line, reflecting typical clinical positioning scenarios such as chin-down and chin-up maneuvers, as well as the operational ranges of CT systems equipped with gantry tilt functionality. Although not all CT scanners currently possess gantry-tilting mechanisms, similar beam incident angles can be effectively reproduced in clinical practice through positioning of the patient (e.g., adjusting head flexion or extension). Therefore, the tilt conditions investigated in this study were considered representative of a broad range of clinically feasible configurations.

In contrast, the present study employed a rigid phantom that did not account for the positional shifts of soft tissues or internal organs associated with changes in patient posture. The phantom was fixed in a mild chin-up position; however, in clinical settings, the actual beam incidence angle and scatter conditions may vary depending on individual differences in posture, range of motion, and the presence of dental materials. Consequently, the dose distributions for organs located in the neck and upper torso, such as the thyroid gland and cervical bone marrow, may be influenced by patient positioning, and caution is warranted when extrapolating these results to clinical applications. Nonetheless, for intracranial organs, the anatomical displacement due to postural variation is generally minimal, supporting the validity of the present dose assessment method. Furthermore, organs within the head, such as the brain, lens, eyeballs, salivary glands, and oral mucosa, are less susceptible to positional changes, and the dose distributions obtained in this study can be directly applied in a clinical context.

Moreover, the phantom used in this study represents a standard adult head model and does not account for individual anatomical variability among patients or technical differences across CT systems. To enhance the clinical applicability of the findings, future studies should include validations using actual patient data, as well as assessments of protocol reproducibility and standardization across multiple institutions and CT devices.

In addition, the dose reduction effects achievable through gantry angulation and scan range adjustments depend on factors such as the institutional workflow, CT system specifications, and imaging objectives. Therefore, clinical studies are required to clarify the practical feasibility and limitations of these techniques in real-world settings. In particular, extending the evaluation to anatomically and physiologically diverse populations such as pediatric and elderly patients is warranted.

This study demonstrates that appropriate adjustment of the gantry tilt angles and optimization of the scan range are effective in reducing radiation doses to multiple organs, including the lens. Furthermore, three-dimensional visualization and analysis of the dose distribution using Monte Carlo simulations provided insights that could not be obtained through conventional physical phantom experiments, highlighting their potential as powerful tools for radiation protection and protocol optimization. It is essential to validate these findings through physical measurements, clinical evaluations, and multicenter collaborative studies, thereby translating them into practical dose reduction strategies in clinical settings.

## Conclusion

In this study, we conducted a detailed evaluation of how gantry tilt angles and scan range settings affect the radiation dose to radiosensitive organs, including the eye lens, as well as the effective dose during head CT examinations using high-precision Monte Carlo simulations with PHITS.

Our analysis revealed that applying an upward gantry tilt of +5˚ or greater from the OM line could reduce the lens dose by up to 87%. Even under vertical irradiation, the alignment of highly attenuating structures, such as the skin and skull, along the x-ray beam path significantly suppressed lens exposure.

Furthermore, for other radiosensitive organs such as the salivary glands, thyroid gland, and oral mucosa, both the gantry angle and scan range settings had a substantial impact on the organ dose. These findings underscore the importance of scan protocol design for optimizing both local and whole-body radiation exposure. Analysis of the effective dose indicated that the inclusion of radiosensitive organs in the scan range was a major determinant of the overall exposure.

Additionally, the approach used in this study enabled the visualization of slice-level dose gradients and spatial heterogeneity, which are features that are difficult to capture using conventional physical phantoms or simplified dose estimation tools. This highlights the potential of simulation methodology as a powerful evaluation tool for radiation protection.

Future efforts should focus on experimental validation, adaptation to different age groups and imaging systems, and multi-institutional standardization to further advance the clinical implementation of dose reduction strategies.

## Data Availability

The datasets generated and/or analyzed during the current study are not publicly available but may be made available from the corresponding author upon reasonable request and with appropriate justification.
